# Changing expression of vertebrate immunity genes in an anthropogenic environment: a controlled experiment

**DOI:** 10.1186/s12862-016-0751-8

**Published:** 2016-09-01

**Authors:** Pascal I. Hablützel, Martha Brown, Ida M. Friberg, Joseph A. Jackson

**Affiliations:** 1 IBERS, Aberystwyth University, Aberystwyth, SY23 3DA UK; 2 School of Life and Environmental Sciences, University of Salford, Salford, M5 4WT UK

**Keywords:** Anthropogenic habitats, Gene expression, Vertebrate, Immunity, Immunoregulation, Seasonality

## Abstract

**Background:**

The effect of anthropogenic environments on the function of the vertebrate immune system is a problem of general importance. For example, it relates to the increasing rates of immunologically-based disease in modern human populations and to the desirability of identifying optimal immune function in domesticated animals. Despite this importance, our present understanding is compromised by a deficit of experimental studies that make adequately matched comparisons between wild and captive vertebrates.

**Results:**

We transferred post-larval fishes (three-spined sticklebacks), collected in the wild, to an anthropogenic (captive) environment. We then monitored, over 11 months, how the systemic expression of immunity genes changed in comparison to cohort-matched wild individuals in the originator population (total *n* = 299). We found that a range of innate (*lyz*, *defbl2*, *il1r*-like, *tbk1*) and adaptive (*cd8a*, *igmh*) immunity genes were up-regulated in captivity, accompanied by an increase in expression of the antioxidant enzyme, *gpx4a*. For some genes previously known to show seasonality in the wild, this appeared to be reduced in captive fishes. Captive fishes tended to express immunity genes, including *igzh*, *foxp3b*, *lyz*, *defbl2*, and *il1r*-like, more variably. Furthermore, although gene co-expression patterns (analyzed through gene-by-gene correlations and mutual information theory based networks) shared common structure in wild and captive fishes, there was also significant divergence. For one gene in particular, *defbl2*, high expression was associated with adverse health outcomes in captive fishes.

**Conclusion:**

Taken together, these results demonstrate widespread regulatory changes in the immune system in captive populations, and that the expression of immunity genes is more constrained in the wild. An increase in constitutive systemic immune activity, such as we observed here, may alter the risk of immunopathology and contribute to variance in health in vertebrate populations exposed to anthropogenic environments.

**Electronic supplementary material:**

The online version of this article (doi:10.1186/s12862-016-0751-8) contains supplementary material, which is available to authorized users.

## Background

During the transition between natural and anthropogenic environments the vertebrate immune system faces combinations of conditions unlike those it evolved to deal with. This is known to result in functional changes [1, 2] and what these changes are, and how and why they occur, is a key problem. There is a direct parallel to health in humans inhabiting relatively anthropogenic settings (for example, higher income countries), where an increasing burden of illness results from non-infectious diseases with inflammatory origins [3–5]. There is an equal relevance to domesticated animals or wild animals occupying urbanized habitats, where the immune system also functions (or malfunctions) under environmental conditions very different to those in nature.

Despite this background, research comparing immune function in the wild to in artificial habitats is in its early stages. The main body of existing work, comparing wild rodents with laboratory counterparts, suggests increased immunological activation in wild animals [1, 6–8], which may result from a greater exposure to infection in nature. Some responses to stimulation may be more intense and variably expressed in wild rodents [6, 7, 9], whilst other responses may be attenuated [9]. Although these results are of great interest, the laboratory rodent models used as the basis for comparison bring with them aspects that may be unrepresentative of the real-world problem. Thus inbred mouse lines, unlike humans and domesticated animals, are genetically homogenous [10] and even outbred stocks may show restricted genetic variability [11]. Furthermore laboratory rodents are maintained under extremely benign and pathogen-free conditions, whereas humans and domesticated animals still encounter many infections and environmental insults, albeit that these are different to those occurring in nature [12]. Moreover, the singular genealogies of laboratory rodents make them difficult to compare directly with wild counterparts, even leaving the effects of inbreeding aside. Thus most laboratory stocks and lines have been in captivity for very many generations and are thus distant from the originator population, if this is identifiable at all. And they will often have been generated through arbitrary crosses [10, 11, 13, 14], resulting in haplotypes unrepresentative of those seen in nature. Hence complex genetic influences confound any comparison made to wild animals, leading to a basic lack of experimental control and uncertainty in interpretation.

In order to understand how loss of natural environment modifies the immune system it will be informative to study the immunophenotypic trajectory of wild animals newly acclimatized to anthropogenic conditions, with matched *in situ* controls in the wild [3, 12]. Importantly, this allows effects due to plasticity and to loss of on-going natural selection in the wild to be studied, unconfounded by long term effects of selection and breeding patterns within the anthropogenic environment. Whilst the latter processes are important in animal domestication, they are a separate issue that is not considered here. Furthermore, it should be noted that selection within the anthropogenic environment is unlikely to explain recent upwards trends in human immunopathologies, given their historical context [3, 12]. These are more likely driven by relatively recent environmental changes, making the focus of the present study of particular relevance.

To provide one case study of the type of acclimatization described above we focussed on the 3-spined stickleback (*Gasterosteus aculeatus*), a species that is accessible and much-studied in the wild [15], that easily acclimates to captivity, and that has an annotated whole genome [16], facilitating post-genomic studies. In the same way that other teleosts, such as zebrafish and medaka, are increasingly used to study disease processes relevant to mammalian health [17], the 3-spined stickleback – because it contains all of the central elements of adaptive immunity [18] - has a general comparative relevance for immunity in other vertebrates.

We transplanted post-larval fishes from a natural habitat to replicated artificial mesocosm habitats and, following anthelmintic treatment of the transplanted individuals, synchronously monitored both wild and transplanted (captive) cohorts through time. This study design embodies a general scenario typical of anthropogenic environments: where parasite exposure is reduced through anthelmintic treatment and curtailment of transmission [19], where bacterial exposures are altered due to artificial diets [20] and substrates [21], and where environmental stressors are different to in the wild due to plentiful food, altered density and social interaction, confined spatial ranges, absence of predation, and altered microclimate and chemical exposures. Our central aim here, though, is not necessarily to dissect the relative contributions of all these influences, but rather to generate a representative scenario and consider the immunological consequences and their health correlates.

As immunological readouts from our experiment we consider changes in the expression of a representative panel of conserved vertebrate immunity genes [22] in whole-fish mRNA pools [23]. In using this whole-organism measurement approach we kept in mind the wide dispersal of the teleost immune system in different tissues [23] and aimed to achieve a holistic metric of immune activity – averaging across the entire immune system and all the tissues of the body. Such a metric is arguably more relevant to the general risk of systemic immunopathology, in comparison to a narrow, arbitrarily chosen focus on a single tissue or cell population within a tissue. Reductionist measurements of the latter type could be unrepresentative at the organism level (as so much is necessarily left unmeasured) and are in danger of reporting cellular trafficking between anatomical compartments, rather than overall levels of systemic activity.

This is the first study that we know of to carry out a closely matched immunological comparison of wild and captive vertebrates (i.e., where the individuals are ontogenetically matched and the results not clouded by differing genealogical histories in the study groups). We use our measurements to ask how do individual immune expression profiles vary between the wild and captivity and do certain immune expression profiles in captivity lead to adverse individual health outcomes?

## Methods

### Experimental design

Our experiment synchronously compared gene expression in wild lacustrine fishes with that in age-class-matched fishes from the same population that had been transferred to a representative anthropogenic habitat. This comparison was extended through time from the point when juvenile wild-caught fish had acclimatized to the anthropogenic habitat to a time when the wild cohort became scarce in nature.

For the anthropogenic habitat, twelve 300 L mesocosm tanks (52.4151°, −4.0670°), arranged in a 3 × 4 array, were evenly stocked with 480 postlarval sticklebacks taken from a lake habitat in mid Wales (52.3599°, −3.8773°) in July-August 2013. The mesocosms constituted two re-circulating systems, with 6 tanks in each. Within each re-circulating system water was re-circulated (3310 L h^−1^) by a pump (Blagdon, MDP3500) via a 90 L biological filter in a header tank. A manipulation of temperature was carried out within the mesocosm array that allowed the effects of thermal variation to be assessed independent of the comparison between wild and mesocosm habitats (as temperature variation might sometimes be confounded with these habitats due to natural climatic variation). Thus, one system was run at ambient temperature and the other heated to 2 °C above ambient temperature from the first experimental sampling point. Temperature in the heated system was maintained by 300 W heaters controlled by digital thermostats (1 per heated tank) with 0.1 °C sensitivity. For temperature control purposes each heated tank was paired to an adjacent unheated tank, with both providing thermistor feeds to the associated digital thermostat. Trials within the tank microenvironments showed that flow rates were sufficient to disperse temperature gradients around heaters within tanks. Tanks from each system were interspersed (alternating rows of 3 heated or 3 un-heated tanks across the array), to reduce positional effects as far as possible. Each tank contained standardized environmental enrichment (plastic aquarium plants) and a layer of light coloured gravel.

Following capture, wild postlarval fishes were subjected to 2 consecutive anthelmintic praziquantel treatments (24 h at 4 mg l^−1^; FlukeSolve, Fish Treatment Limited), separated by four days, following manufacturer’s recommendations. Of the common infections present in the wild population (based on 510 fish monitored between July 2013 and October 2015), these treatments completely removed *Gyrodactylus* sp. and a diplostomatid digenean species (the latter infecting the retinal layer of the eye), but had no measurable effect on *Schistocephalus solidus* worms or on ectocommensal trichodinids and epistylids. Prior to the commencement of the experiment in October, fishes were acclimatized for 4–6 weeks within the mesocosm system. Salinity in the system was routinely maintained at approximately 1 % (10 g L^−1^) as a prophylactic measure to suppress opportunistic microbial infections. Nitrite and nitrate levels (Tropic Marin Nitrite-Nitrate test) were continuously monitored throughout the experiment and remedial water changes carried out when nitrite levels rose above 0.02 mg L^−1^. Animals were fed daily on standard (per mesocosm) rations of frozen chironomid larvae (“bloodworm”), occasionally supplemented with frozen cladocerans (Tropical Marine Centre). Temperature in each tank was logged every 5 min, to a reading resolution ≤ 0.05 °C, throughout the experiment by Tinytag radio temperature loggers (TGRF-3024) networked through a Tinytag Radio system. In the field, temperatures were logged every 5 min by a Tinytag Aquatic 2 (TG-4100) data logger to a reading resolution ≤ 0.01 °C. Mean temperatures tended to be slightly higher (average difference < 1 °C) in the un-heated mesocosms than in the natural lake habitat (Additional file 1: Fig. S1a). Due to monthly sampling, individual density in the mesocosms fell from ~ 0.13 individuals L^−1^ at the start of the study period to ~ 0.028 individuals L^−1^ at the end. Reduction in individual density though, tended to be compensated by individual growth, with biomass density ranging within relatively narrow limits: from 0.013 to 0.027 g L^−1^ (Additional file 1: Fig. S1b). As the densities that we employed were relatively very low, they would have reduced the negative biological effects of crowding. At the same time, sufficient numbers of fish remained in each tank to allow these to aggregate in large groups and undergo social interactions. This scenario may have limited any influence of density variation on immunity (via crowding [24, 25] or social [26] mechanisms), although such effects cannot be eliminated. Notwithstanding, as density effects would not be biologically comparable in the field and mesocosms (wild individuals being able to range over much larger distances and interact with a much larger total population), we did not include density estimates in the analyses below. Instead we expected any between-habitat density-related differences to emerge in the habitat and habitat x time terms of statistical models. As considered above, altered density and constraints on movement and social interaction are just one of the many environmental components varying between natural and anthropogenic habitats - and it is beyond the scope of the present study to identify and fully dissect the influence of each of these components.

Animals were sampled monthly, on the same day, between October 2013 and August 2014: 10 animals month^−1^ in the originator lake habitat and 20 animals month^−1^ in the mesocosms (10 animals each from the heated and un-heated systems, drawing animals evenly from amongst different tanks). For the mesocosms, some additional monthly sampling (at the same sampling points as above) was carried out to provide (unused) spare capacity for the present study. Monthly samples from the wild and from mesocosms would have been approximately matched for age because they originated in the same annual recruitment cohort and this would have ceased to recruit new individuals soon after the last mesocosm stock were collected in the field. Although sticklebacks may breed across the spring and summer in the lake habitat, leading to a range of individual ages within the year cohort, this variation would likely have been distributed similarly within individual monthly samples (of 10 or 20 fish). Furthermore, the inclusion of length (a partial surrogate for age [23]) in statistical models (see below) will have additionally adjusted for age variation between individual fishes.

### Animal handling and nucleic acids preparation

All animal maintenance and sampling of animals in the field followed U.K. Home Office regulations and local (Aberystwyth University) ethical approval procedures. Mesocosm work involved only manipulations fully compatible with routine captive fish husbandry (where the aim is to maintain a healthy stock) and was carried out in consultation with the HO inspectorate. Sampling occurred at standardized times of day, 09.00–13.00 h (UTC), and within a 2 h window at each sampling occasion. Sticklebacks were captured individually using a dip net and immediately killed by concussion and de-cerebration and stored in *RNAlater*™ RNA stabilization solution (ThermoFisher Scientific) according to manufacturer’s instructions (>5 volumes of stabilization solution; ≤ 0.5 cm solid tissue thickness along smallest dimension; a ventral incision was made to the abdominal cavity of each specimen to aid penetrance of stabilization solution). Samples were transferred to 4 °C overnight and then to −80 °C for long-term storage. Immediately prior to RNA extraction, sticklebacks were thawed at 4 °C, dabbed dry with tissue, and examined for *Schistocephalus* infection under a dissecting microscope (via a ventral incision). Fish weight (mg, minus the weight of any *Schistocephalus* infection) and standard length (mm) were recorded. RNA from whole fishes was extracted using the *Isolate II RNA mini kit* (Bioline): whole individual fishes were homogenized in lysis buffer using a 5 mm stainless steel bead (Qiagen, 69989) in a Qiagen TissueLyser LT system and a standard aliquot of the homogenate (diluted in lysis buffer to be equivalent to 11 mg of tissue) passed through the manufacturer-recommended protocol. Trials with whole fish preserved as above indicated that high RNA yields of good quality were consistently obtained from internal organs (spleen and head kidney), providing evidence of effective penetrance of RNA stabilization solution to the deep tissues.

### Choice of target genes

Target genes were partly selected to represent a diversity of immunological pathways and partly to include those immune-associated genes determined to have high environmentally-related variability in a previous transcriptomic (RNAseq) study of sticklebacks in mid Wales [23]. Adaptive responses were represented by the T-cell receptor co-receptor alpha chain from cytotoxic T-cells (*cd8a*) [27], the heavy chains of immunoglobulins M (*igmh*) and Z (*igzh*, see Gambón-Deza et al. [28]), the pro-inflammatory T-helper cell type 1 cytokine interleukin 12 (*il12ba*) [29], the pro-inflammatory T-helper cell type 17 cytokine interleukin 17 [30], the T-helper cell type 2 cytokine interleukin 4 (*il4*, see Ohtani et al., 2008 [31]), the regulatory T-cell transcription factor forkhead box P3 (*foxp3b*) [32] and a gene encoding a calcium channel known to be necessary for T-cell activation and proliferation in mammals (*orai1*) [33]. Innate defences with direct activities against microbes, that may be expressed constitutively or inducibly following the stimulation of innate cells, were represented by the antimicrobial peptide beta defensin (*defbl2*,) [34, 35] and the bacteriolytic enzyme lysozyme C (*lyz*) [36]. Innate inflammatory responses were represented by genes involved in toll-like receptor (TLR) (*tirap*, *tbk1*) [37, 38] and interleukin 1 (IL-1) family signalling (*il1r*-like) [39]. We also measured the expression of the anti-oxidative enzyme, glutathione peroxidise 4a (*gpx4a*) [40], to reflect responses to oxidative stress that might drive, or be driven by, inflammation [41]. Importantly, because of the elevated salt concentrations maintained in the mesocosm systems, none of these loci are present in a previously reported list of 1844 stickleback salt responsive genes [42].

### Q-PCR gene expression measurements

Quantitative real-time PCR (Q-PCR) gene expression measurements were carried out in a 2-step format with SYBR Green chemistry. Primer sets (Table 1) used to assay target and endogenous control genes featured exon-spanning primers (except in the case of *tirap*, a single exon gene) and were determined to be 100 ± 10 % efficient under reaction conditions. Endogenous control genes, *yipf4* and *acvr1l*, were previously selected as an optimally stable pairing for whole-fish analyses by the *NormFinder* algorithm [43] applied to RNAseq expression data for 36 wild fishes sampled at different sites in summer and winter [23]. As for the target genes listed above, both endogenous control genes are absent from a reported list of stickleback salt-responsive genes [42]. RNA samples were DNase treated and quantified spectrophotometrically (NanoDrop 2000) prior to conversion to cDNA with the *High Capacity RNA-to-cDNA Kit* (Life technologies) at the kit’s maximum capacity. A proportion of samples were also processed with reverse-transcriptase negative controls. Each cDNA product was diluted 1:20 prior to assaying. Samples were run in a 384-well plate format on a Life technologies QuantStudio 12 K flex real-time PCR system using *SensiFAST SYBR® Lo-ROX* mix (Bioline) and with the instrument manufacturer’s default fast cycle settings and recommended reaction concentrations scaled to 10 μl reactions. All assay plates were pipetted by an Eppendorf epMotion M5073 robot using a standard programme and layout. In addition to unknown samples (run in duplicate), each assay plate included a calibrator sample (run in triplicate) and no-template control wells (for every gene). For each sample, all target and endogenous control genes were run within a single plate and samples from different sampling units (time points and origins) were balanced across plates. Melting curves (run at instrument default settings) were examined for each assay well to assess the possibility of non-specific amplification. Relative expression (indexed to the calibrator sample and based on mean values for the technical replicates) was calculated using the ΔΔCt method. The calibrator sample was created by pooling cDNA aliquots from all individual samples.

**Table 1:** Primers used for quantitative real-time PCR (Q-PCR) measurements

Gene	Ensembl gene number (or other source of sequence)	Primers
*cd8a*	ENSGACG00000008945	F - CCACCCTGTACTGCAATCGA
		R - CCGCCTGCTGTTTTCTTTTG
*foxp3b*	ENSGACG00000012777	F - TCTGAACACAGTCATGGGGAGA
		R - CCAGGATGAGCTGACTTTCCA
*orai1*	ENSGACG00000011865	F - GCACCTCGGCTCTGTTGTC
		R - CCATGAGGGCGAAGAGGTGTA
*tbk1*	ENSGACG00000000607	F - AGACGGAGCAGCTGTTCGA
		R - GCATATCTCATCATATCTGACGACAT
*il1r*-like	ENSGACG00000001328	F - GAACGCGAGAACTGCAAGAAC
		R - GGGACGCTGGTGAAGTTGAA
*igmh*	ENSGACG00000012799	F - GGAGGCAAAGGACGCTACTTT
		R - AACCACATTTGGCCTTTGGA
*igzh*	Gambón-Deza et al., 2010 [28]	F - TCAACAAAGGAAATGAACCAAAA
		R - TCTTCCTCTGGGAGGACGTG
*il12ba*	ENSGACG00000018453	F - TTCATCAAAGCTTGGCGTT
		R - CCGCCGTCCACAGAACAC
*il17*	ENSGACG00000001921	F - GGGCCTACAGGATCTCCTACG
		R - GCCCCTGCACAGGCAGTA
*il4*	Ohtani et al., 2008 [31]	F - CCAAAATCAAACCTGTGCAGTGT
		R - CGAGAAGTCGCGGAATCTGT
*defbl2*	ENSGACG00000020700	F - TGCAGACGGTTCTGCTATGC
		R - GGCACAGCACCTGTATCGTC
*lyz*	ENSGACG00000018290	F - TGTCAGAGTGGCAATCAATTGTG
		R - CCCACCCAGGCTCTCATG
*tirap*	ENSGACG00000006557	F - GGGGCGCCATTTCTACAGA
		R - TGCATCATGTACTGGCACC
*gpx4a*	ENSGACG00000013272	F - CCAGGAACCCGGCAATG
		R - GAACCGAGCGTTGTAAGGAC
*acvr1l*	ENSGACG00000010017	F - CACTTTAGCGGAGCTGTTGGA
		R – AGAAAAGGAAGTCCGGAACCA
*yipf4*	ENSGACG00000002189	F – CCCTCAAACGGAGACTTTACGT
		R - GGTGCCGCTGAGCTCTTC

Primers used for quantitative real-time PCR (Q-PCR) measurements

### Data analysis

Only wild fishes from the same year class as the mesocosm fishes were included in analyses. Our analyzed dataset consisted of 299 fishes, 84 from the wild and 215 from the mesocosms. Of these, 4.6 % overall (1 wild fish and 13 mesocosm fishes) had one or more missing measurements and thus are omitted from some or all analyses. An initial test of the hypothesis that habitat affected the expression of immunity genes was carried out using multivariate analysis of variance (MANOVA), with all immune gene expression variables as the responses, and with habitat, sex, *Schistocephalus* infection (present/absent) and time (month) as factors and body length, body condition and mean temperature in the 14 days before sampling as covariates. Inclusion of body length in the model allows for variation in immune expression due to age (for which body length is a substantial surrogate [23]) and general size, including the possibility of tissue allometry affecting whole fish measurements [23]. Body condition was derived as the residuals from a quadratic regression of body weight on body length [44] (and there were no linear associations of the weight residuals with sampling time in either habitat, suggesting that the use of the residuals as a condition index was not biased by ontogenetic stage). Following a significant result in the MANOVA, the effects of habitat on the expression of individual immunity genes, and also *gpx4a*, were considered using general linear models (LMs) of analogous structure to the MANOVA (and allowing variation in the set of explanatory terms used for each gene expression response; see next). Because temperature was partially confounded with habitat (Additional file 1: Fig. S1a), and did not affect many of the genes examined across the relatively large (2 °C) thermal manipulation in the mesocosms, the MANOVA described above may have been conservative in detecting habitat effects. Thus for the LMs we only included a temperature covariate for those genes where we found a significant effect of temperature in the mesocosms. The effect of *Schistocephalus* infection and temperature within the mesocosm environment was tested in LMs containing factors for sex, *Schistocephalus* infection, time and temperature treatment (ambient/+ 2 °C) and covariates for body length and body condition. The association of individual gene expression variables with body condition (weight residuals, see above) in mesocosm fishes was also tested in LMs, in this case additionally containing factors for sex, *Schistocephalus* infection, time and temperature treatment. In all cases gene expression data were log_10_ transformed before analysis by MANOVA or LM. Principal components analysis (PCA) was used to ordinate gene expression measurements from individual fishes in order to identify habitat-specific patterns of variation. Differences in the overall variability of untransformed individual gene expression variables between the field and the mesocosms were tested using Levene’s test for equality of variances. MANOVA, LMs, PCA and Levene’s test were implemented in Minitab version 16.2.2. For co-expression analyses we used gene expression residuals from LMs (with terms for sex, length, body condition and *Schistocephalus* infection) in order to adjust for confounding variation. We initially constructed Pearson correlation matrices from the residuals and tested for significant structure in individual matrices (null = an identity matrix) using a Steiger test [45], and for differences between matrices with a Jennrich test [46] (using the *R* package *psych*). We tested for similarity in structure between matrices with a Mantel test [47, 48] (using the *R* package *ape*). We also used the residuals to construct co-expression networks with the information-theory (mutual information, MI) based algorithm *ARACNe2* (Algorithm for the construction of accurate cellular networks) [49, 50], which takes non-linear associations into account. For this analysis we set all genes as hubs and constructed networks using the adaptive partitioning algorithm, estimating the MI threshold by a pre-processing run. *P* threshold was set at 1 × 10^−7^ and the data processing inequality at zero. Networks were bootstrapped (2000 resamples; significance cut-off for reported edges, *P* = 1.0 × 10^−6^). *Cytoscape* 2.8 was employed to visualize the networks (using arbitrary force-directed layouts) and to calculate network intersections (*Network Analyzer* plugin).

## Results

### A range of innate and adaptive immunity genes are up-regulated in captivity and this is, in part, associated with a loss of seasonality

**Fig. 1:**
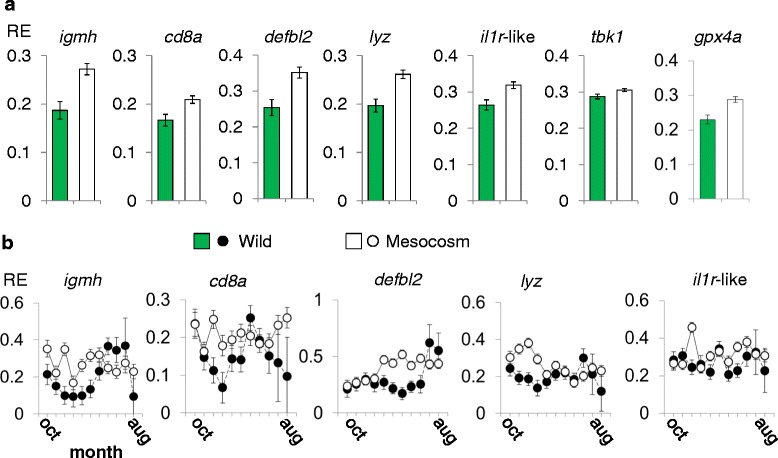
Elevated expression of diverse immunity genes in anthropogenic compared to wild habitats. **a** Bar charts showing significant main effects of habitat (Additional file 2: Table S1); bars represent predicted relative expression (RE) ± 1 standard error from confounder-adjusted general linear models (LMs). **b** Plots for RE showing interaction between habitat and time; for *igmh* and *cd8a* there is a diminution of summer-biased seasonal patterns in mesocosm fishes; points represent predicted RE ± 1 standard error from LMs. Note that July and August time points for wild fishes are supported by relatively few data

Fishes reared in captivity or taken from the wild showed similar, and extensively overlapping, biometric characteristics (Additional file 1: Fig. S1c, d). An initial overall confounder-adjusted MANOVA test for expression in 13 immunity genes revealed a highly significant difference (main effect) between wild and mesocosm fishes (*F*_14,256_ = 3.72, *P* <0.0005). To dissect this result further we then examined each of the individual variables in confounder-adjusted general linear models (LMs) (Fig. 1a; Additional file 2: Table S1), finding significant main effects for *igmh*, *cd8a*, *defbl2*, *lyz*, *il1r*-like and *tbk1*. In all of these cases, expression was very significantly up-regulated in the mesocosms. We also considered the antioxidant enzyme, *gpx4a*, and found that this too was very strongly up-regulated in the mesocosm fishes. Furthermore, this gene co-varied positively with most immunity genes, consistent with a link to oxidative stress that might drive, or be driven by, immune responses. Thus log-transformed *gpx4a* was positively associated with the major axis of a log scale PCA of immunity genes in both habitats (lake *r* = 0.59, P <0.0005; mesocosms *r* = 0.55, *P* <0.0001), where this axis predominantly represented positive covariation, and it was individually positively associated (*P* <0.05) with 6 out of 13 log-transformed immunity genes in lake fishes and 12 out of 13 genes in mesocosm fishes.

For genes with significant main effects for habitat we also examined all two-way interactions involving habitat (except for the interaction with temperature, where this was included in the model). This revealed that in most cases there was a complex interaction with time (Fig. 1b; Additional file 2: Table S1), though the form of his interaction was not consistent across genes, with relatively elevated expression in the mesocosms occurring in the autumn and winter (*igmh*, *cd8a*, *lyz*), the winter and spring (*defbl2*) or without a clear pattern (*il1r*-like). In some cases, if data for July and August are put aside due to the small number of wild fishes sampled, peak average expression in the mesocosms reached higher than it ever did in the wild (*lyz*, *il1r*-like*, defbl2,* Fig. 1b). On the other hand, for *cd8a* and *igmh* (genes previously known to show summer-biased expression in the wild [23]) mean expression was more consistent in mesocosm fishes, diminishing any seasonal pattern. Thus similar high expression levels of *cd8a* and *igmh* occurred in the mesocosms and the wild during summer, but mesocosm fishes maintained high expression during winter periods when this was reduced in wild fishes. Additional to the interactions with time, there was an interaction of habitat with length (*F*_1,259_ = 10.09, *P* = 0.002) for *defbl2*, with a trend for increased expression in smaller mesocosm fishes.

### Captive animals have altered patterns of gene co-expression

As the regulatory influence of genes on other genes may be reflected in patterns of co-expression, we searched for differences in such patterns that might reveal regulatory changes in the immune systems of wild and captive fishes. We based this analysis on fishes from un-heated mesocosms, so that temperature conditions were similar between the two samples (<1 °C average difference) and there were approximately equal sample sizes in the two groups. In order to remove across-site biases due to individual host variables, we took residuals from LMs including terms for sex, length, body condition and *Schistocephalus* infection.

**Fig. 2:**
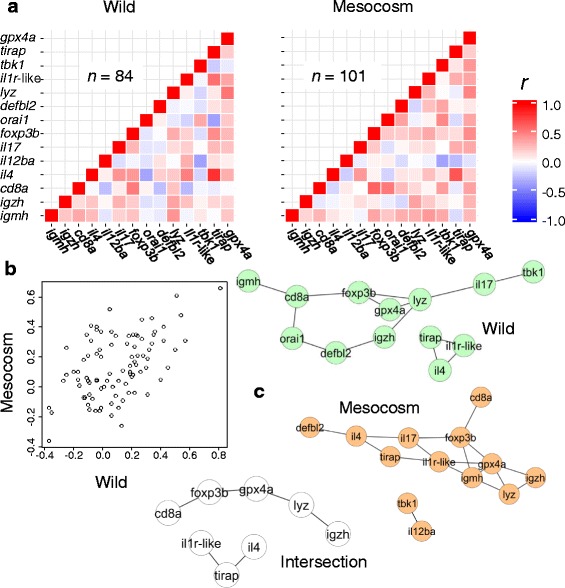
Altered gene co-expression patterns in anthropogenic compared to wild habitats. **a** Gene expression correlation matrices (Pearson, *r*) were significantly different in wild and mesocosm fishes (Jennrich test, *P* = 6.89 × 10^−13^) but also retained similar structure (Mantel test, *P* = 0.001). **b** Scatter of pair-wise correlation coefficients for mesocosm and wild fishes: the wild coefficients explain 27 % of the variation in mesocosm coefficients in a linear regression. **c** Gene co-expression networks for wild and fishes constructed using the *ARACNe* algorithm, and the intersection of these networks showing shared edges (shared statistical associations between genes)

Initially considering pair-wise (residual) gene expression correlation matrices for the two sites, we found that both were strongly structured (Steiger test; wild, *P* = 2.7 × 10^−62^; mesocosm, *P* = 1.2 × 10^−79^). The matrices differed significantly (Jennrich test, *P* = 6.89 × 10^−13^), but retained some similarity (Mantel test, *P* = 0.001) (Fig. 2a), with correlation coefficients from wild fishes explaining 27 % of the total variation in coefficients from captive fishes in a linear regression (Fig. 2b).

We then compared (residual) gene co-expression networks for wild and captive fish constructed using the information theory-based algorithm *ARACNe*, which takes into account non-linear associations between the individual variables (Fig. 2*c*). Some edges (statistical links between genes) were identical in both networks (43 % shared in wild fishes and 38 % in mesocosm fishes) but there were also a large number of differences. These included the absence of *orai1*, a gene predicted to be involved in seasonal immune function [23], from the mesocosm network. Also the antioxidant enzyme *gpx4a* showed co-expression with more genes in the mesocosm network.

### Only *defbl2* expression was associated with individual condition in captive fishes

We considered how the genes up-regulated in captivity might be associated with individual condition (weight residual, see Material and methods) specifically within the mescosm environment. We found only a (negative) effect for *defbl2* (*F*_1,192_ = 9.49, *P* = 0.002).

### The expression of immunity genes is more variable in captive fishes

**Fig. 3:**
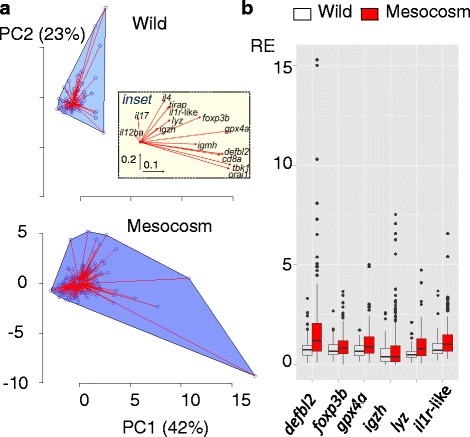
More variable expression of immunity genes in anthropogenic compared to wild habitats. **a** Bagplots showing, for wild (above) and all mesocosm (below) fishes, the distribution of individual scores along the 2 major axes of an overall principal components analysis (PCA) of immunity genes; plots show outer hull (a minimally enclosing convex polygon) containing all points, individual points and lines joining individual points to the group centroid. There was a significant difference in variance between wild and mesocosm fishes scores along PC1 (*P* = 0.024; *P* = 0.016 if including only mesocosm fishes from un-heated tanks). PC1 accounted for 42 % of total variation and PC2 23 %. Inset in upper panel shows a biplot of variable loadings on PC1 and PC2; vectors emanate from the origin and the axis scales (x : PC1, y: PC2) are indicated in the bottom left corner of the inset. PC1 represented positive covariation in the majority of genes (mostly substantial loadings of the same sign), whilst PC2 represented contrasts between genes (substantial loadings of variable sign), some of which might be explained by opposing seasonal patterns in the wild [23] and the diminution of these patterns in the mesocosms. **b** Box-and-whisker plot of individual expression values for genes with significant differences in expression variance between wild and mesocosm fishes (Additional file 3: Table S2). Box shows interquartile range (IQR) and median (line); whiskers extend to most distant observations within a 1.5 × IQR distance of the IQR; points show outlying values (>1.5 × IQR distant from IQR)

We also considered the comparative amount of variability in gene expression in the wild and in captivity. Individual scores along the major axis of variation in a PCA of raw (untransformed) data, including all specimens from both habitats, were more variable in the mesocosms (Fig. 3a) (Levene’s test, *P* = 0.024). This major axis (PC1) accounted for 42 % of total variation and principally represented positive covariation in gene expression (i.e., with component loadings mainly of the same sign; see Fig. 3a). Mesocosm fishes extended much farther than wild fish along PC1 in the direction of higher expression values (but not in the other direction), indicating that fishes with high levels of overall immune activity were responsible for the greater variability in the mesocosms. A similar result was obtained (with greater statistical significance), if only individuals from un-heated mesocosm tanks were included in the comparison (*P* = 0.016). For individual genes (Fig. 3b), untransformed expression of *igzh*, *foxp3b*, *lyz, defbl2, il1r*-like and *gpx4a* was significantly more variable in the mesocosms, and this remained the case if only un-heated tanks were included in the comparison (Additional file 3: Table S2). Across this set of heteroscedastic genes (especially *dfbl2* and *igzh*), considerable numbers of mesocosm fishes demonstrated expression values well in excess of that ever seen in wild fishes (Fig. 3b).

### There is no effect of *Schistocephalus* infection on genes up-regulated in the mesocosms

Finally we asked whether a reduced burden of macroparasitic infections might contribute to the immunological changes seen (above) in the mesocosms. Although differences in infection pressures were generally confounded with other mesocosm characteristics, we were able to cleanly assess the effect of one infection, *Schistocephalus*, amongst mesocosm fishes. This infection was refractory to the anthelmintic treatment used at the start of the study and infected a substantial proportion of mesocosm fishes (36 %), providing the basis for comparison on a level playing field. We found that there was no effect of *Schistocephalus* infection on any of the genes up-regulated in captivity.

## Discussion

We found significant up-regulation of aspects of both innate and adaptive immune gene expression in captive (mesocosm) compared to wild fishes. When host and environmental confounders were accounted for, 6 out of 13 (46 %) of the immunity genes examined were differentially expressed, in all cases up-regulated in the mesocosm environment. Of the six up-regulated genes, two are adaptive immunity genes pivotal in cytotoxic T-cell (*cd8a*) and antibody (*igmh*) responses. Of the other up-regulated genes, the bacteriolytic enzyme lysozyme (*lyz*) and the antimicrobial peptide beta defensin (*defbl2*), code for molecules with direct antimicrobial activity and that may be deployed by the host as standing or inducible innate defences. The remaining up-regulated genes (*il1r*-like, *tbk1*) are components of signalling pathways involved in innate inflammation. Although we did not directly observe how these gene expression responses corresponded to functional immune responses, their relevance to functional immune status is supported by the frequently observed correlation between gene expression and infection in sticklebacks [51–54] and many other vertebrates. Furthermore, although complex post-transcriptional kinetics might occur for some individual genes that lead to mRNA and bioactive protein responses of differing sign [22], the concordance in the direction of the above responses is indicative of a broad up-regulation of diverse immune pathways in captivity.

For some of the genes up-regulated in captivity, there was evidence that this was due to a diminution of seasonality. Thus, for two adaptive immunity genes previously known to show summer-biased expression in the wild (*cd8a*, *igmh*) [23], there was a time × habitat interaction in statistical analyses. The form of this interaction was such that maximum average expression values were similar in both habitats, but captive fish tended to maintain high expression during winter periods when expression was seasonally reduced in the wild. In these cases more challenging environmental conditions in winter that lead to a reduction of adaptive immune activity may be the origin of the overall difference [23]. In contrast, for other genes (*defbl2*, *lyz*, *il1r*-like), differing temporal responses occurred that involved higher average expression in captive fishes than was ever recorded in wild fishes. Importantly, as such responses may extend (in some individuals) outside the range seen in nature, and thus outside the parameter space previously operated upon by natural selection, they may uncover maladaptations in immunoregulatory networks and increase the risk of immunopathology [55].

Analysis of inter-individual variability confirmed that the expression of immunity genes was generally less constrained in captive than in wild fishes. A number of genes showed significant differences in the variability of expression in the wild and captivity (*igzh*, *foxp3b*, *lyz, defbl2*, *il1r*-like and *gpx4a*), and in all cases these were more variable in captivity. This increased variability was largely due to some captive individuals with high expression levels. These observations are consistent with a relaxed control of immunological activity in captive fishes, perhaps due to a lack of strong and overriding environmental pressures that tend to affect all individuals in the population (for example seasonal change, or greater motor activity required in foraging and predator avoidance).

When we considered the pattern of co-expression amongst genes, via gene-by-gene correlation or information theory based networks, our analyses revealed a similar core structure in the wild and in captivity, but also significant divergence between the two. For example, *orai1* - a gene we have already implicated in seasonal immune function [23] - was (statistically) linked to other genes within the co-expression network for wild fishes, but not within the network for mesocosm fishes. This further emphasizes a disruption of seasonal responses (see above) as one of the consequences of captivity. Interestingly, expression of the antioxidant enzyme, glutathione peroxidise 4 (*gpx4a*) [40] tended to correlate positively with that of immunity genes in both captive and wild fishes, and was very strongly up-regulated in captive fishes in comparison to wild fishes, suggesting a propensity to track the general level of immune activity. In networks and correlation analyses this gene demonstrated more statistical links to immunity genes in captive than in wild fishes. Taken together, these observations are consistent with captive fishes generating greater anti-oxidant activity to counteract oxidative pressures that might drive, and be driven by, increased inflammation [56]. Alternatively, there may be mechanisms that limit immunological activity according to anti-oxidative capacity [57] (i.e., where anti-oxidative potential is low immune responses are curtailed in order to prevent oxidative self-damage). In this latter regard, one possible link to environmental variation might be through selenium intake in the diet, as this element can be present in limiting quantities in nature and is a cofactor for selenoprotein enzymes (including glutathione peroxidases) involved in anti-oxidative physiology [58, 59].

We also considered whether genes that were up-regulated in captivity were associated with the health status of individual captive fishes, using body condition (weight adjusted for length) as a proxy for health. We found that only *defbl2* was associated, negatively, with body condition. Interestingly this gene also tended to be expressed more strongly in smaller captive fishes (adjusting for body condition), but did not show a similar trend in wild fishes, consistent with a link to growth patterns in captivity. As maximum individual expression levels of *defbl2* in captivity extended well outside those ever seen in the wild, this may be an example of a maladaptive immune response - previously unrefined by natural selection - that results in immunopathology. It is worth noting, furthermore, that high beta defensin gene copy number (which can increase beta defensin expression levels) is known to be associated with immunologically based disease in humans (Chron’s disease and psoriasis) [60].

Our design compared the overall effects of a “complex” of environmental changes, associated with transfer to artificial environments, to the situation in the wild. Although the loss of most parasites was confounded with these general environmental changes, the refractoriness of *Schistocephalus* to anthelmintic treatment meant that the effect of this infection could be considered against the background of domestication conditions. *Schistocephalus* infection, though, did not explain variation in the expression of the immunity genes that were up-regulated in captivity. Thus, if parasite loss is an important agent of immunological change in captivity, then this might only be through a subset of the naturally-occurring assemblage not including *Schistocephalus*. Interestingly, *Schistocephalus*, which occupies a sterile site of infection (the abdominal cavity), may not be able to influence the immune system indirectly through interactions with symbiotic microbiota [61].

A further possibility that should be considered is that selection in the wild resulted in some of the expression differences that we observed. Thus the mesocosms, where mortality was negligible during the course of the experiments, would have experienced a very different selective landscape to in the wild, where there is steady attrition of year cohorts. The present results could in part be consistent, for example, with selection in the wild purging individuals whose expression profiles drift outside a given parameter space. On the other hand, cryptic selection at the point of stocking mesocosm fishes is an unlikely explanation for the patterns that we saw because, rather than occupying a subset of the phenotypic space spanned by wild fishes, mesocosm fishes in fact occupy a much wider phenotypic space.

## Conclusions

In summary, we have conducted a large, carefully controlled experiment comparing immune system expression in the wild and in a representative anthropogenic setting, using ontogenetically synchronized subjects with matched genealogical and environmental origins. Adjusting for the effects of confounder variables, a broad set of vertebrate immunity genes, representing diverse pathways, were up-regulated and expressed more variably in populations in the anthropogenic environment. This was accompanied by changes in patterns of co-expression and an increased signature of anti-oxidative activity (expression of *gpx4a*). The latter trend and the generally strong patterns of co-expression involving *gpx4a*, suggest that immune activity levels are tightly linked to oxidative status. It should be noted that observed changes from the wild state were coincident with the complex of environmental shifts and the altered selective landscape experienced by individuals acclimatising to the anthropogenic environment. However, it was possible to rule out immunological effects of the larval pseudophyllidean cestode *Schistocephalus* as a source of changes and to pinpoint a dampening of seasonal responses as an important effect for some genes. Taken together, our results demonstrate widespread regulatory modulation across the immune system of individuals maintained in an anthropogenic environment. Importantly, our whole-organism measurement approach allows us to infer an increase in systemic immunological activity – extending, in some individuals, into areas of phenotypic space that are rarely occupied by wild animals. The occupation of such exotic phenotypic spaces (as, for example, in the case of beta defensin) may uncover features in immunoregulatory networks that have never been refined by selection and that may result in immunopathology.

## Additional files

Additional file 1: Figure S1. Biometric and environmental variation in the study populations and sites. (PDF 42 kb) Additional file 2: Table S1. Significant effects of habitat and habitat × time on the expression of immune-associated genes. (PDF 322 kb) Additional file 3: Table S2. Differing gene expression variance in wild and mesocosm fishes. (PDF 136 kb)

## Abbreviations

IQR: Interquartile range
LM: General linear model
MANOVA: Multivariate analysis of variance
PCA: Principal components analysis
Q-PCR: Quantitative real-time PCR
RNAseq: High throughput RNA sequencing
