# Supplementary material for: Changing expression of vertebrate immunity genes in an anthropogenic environment: a controlled experiment
Source: BMC Evol Biol. 2016 Sep 1;16(1):175. doi: 10.1186/s12862-016-0751-8 (PMC5009682; doi:10.1186/s12862-016-0751-8)
Supplement: Additional file 1: Figure S1. — Biometric and environmental variation in the study populations and sites. (PDF 42 kb) [file 12862_2016_751_MOESM1_ESM.pdf]

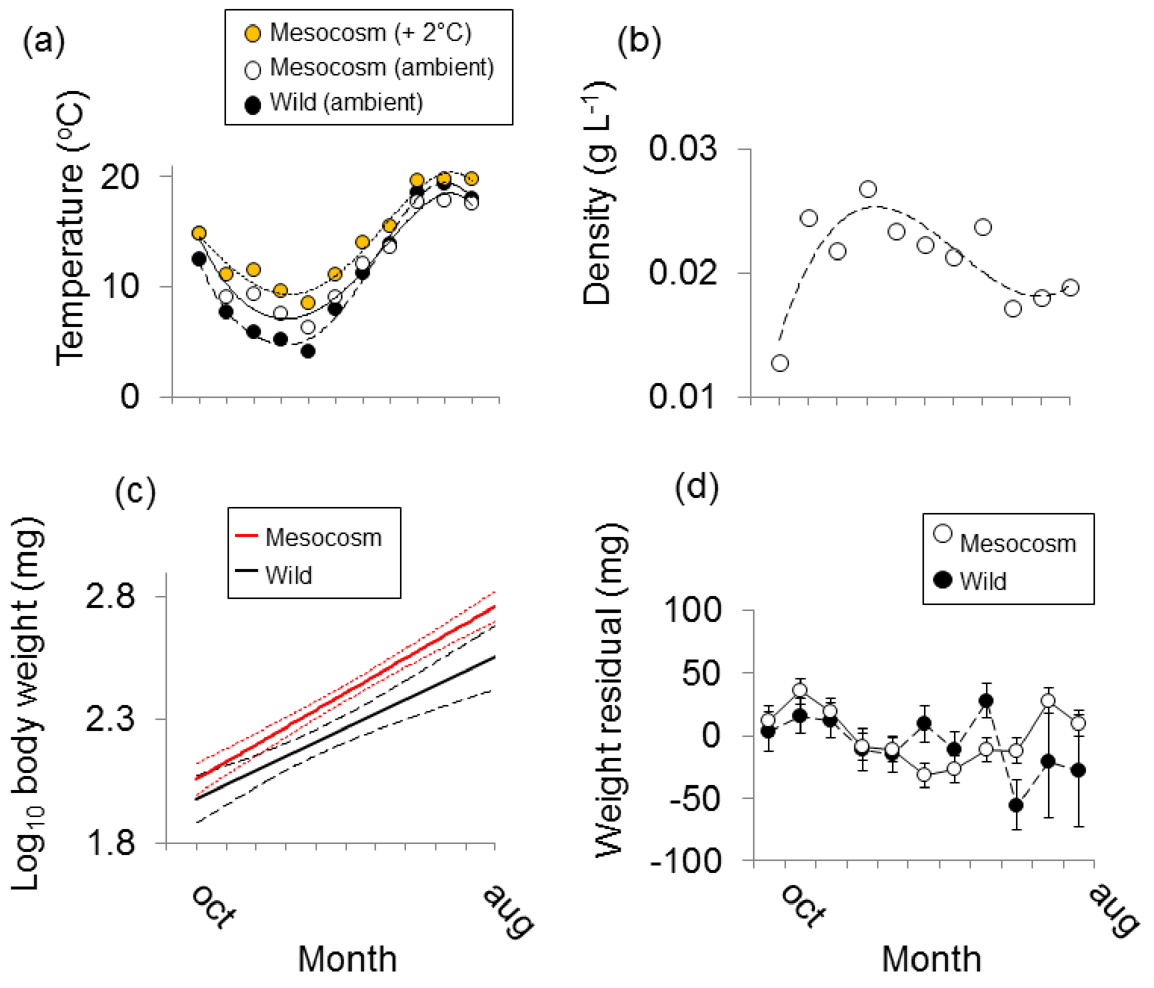

Fig. S1 Biometric and environmental variation in the study populations and sites. (a) Temperature variation in the wild and mesocosm habitats; temperatures tended to be slightly lower in the wild; data plotted are means for the two weeks prior to the monthly experimental sampling point. (b) Plot of estimated biomass density against time in the mesocosm habitats, showing a polynomial trendline; biomass density was relatively low compared to typical values in captive-maintained fishes; there was a peak in January but individual growth tended to compensate for reduction in numbers (due to sampling), limiting the range of variation to ~ 0.01 g L^{-1}. (c) Regression of $\log_{10}$ body weight against time (based on single observations per fish with monthly sampling); although mesocosm fishes were slightly (significantly) larger, they grew at the same overall rate as wild fishes. Plotted data are predictions and 95% confidence intervals from a general linear model (LM) with terms for habitat ($F_{1,293}$ = 18.51, $P < 0.0005$) and time (month specified as a covariate) ($F_{1,293}$ = 206.62, $P < 0.0005$). When habitat × time was added to this model it was non-significant

($F_{1,292}$ = 1.20, P = 0.275) . (d) Plot of predicted body condition (weight residuals from a quadratic regression on length) against time. There was no overall difference in body condition between wild and mesocosm fishes. Body condition declined in the winter months and breeding season in mesocosm fishes, but this trend was less clear in wild fishes. Plotted data are predictions (± 1 standard error) from an LM with terms for habitat ($F_{1,278}$ = 0.64, P = 0.425), time (month specified as a factor) ($F_{10,278}$ = 5.24, P < 0.0005), *Schistocephalus* infection (present/absent) ($F_{1,278}$ = 30.24, P < 0.0005), sex ($F_{1,278}$ = 0.39, P = 0.535) and habitat × time ($F_{10,268}$ = 1.89, P = 0.057) (main effect test results given are for model lacking interaction term).
