# Supplementary material for: Changing expression of vertebrate immunity genes in an anthropogenic environment: a controlled experiment
Source: BMC Evol Biol. 2016 Sep 1;16(1):175. doi: 10.1186/s12862-016-0751-8 (PMC5009682; doi:10.1186/s12862-016-0751-8)
Supplement: Additional file 2: Table S1. — Significant effects of habitat and habitat × time on the expression of immune-associated genes. (PDF 322 kb) [file 12862_2016_751_MOESM2_ESM.pdf]

Table S1*a*. Significant results for the main effect of habitat on the expression of immune-associated genes in general linear models (LMs)[†]

**Gene**	**df1**	**df2**	***F***	***P***
igmh	1	274	15.42	< 0.0005
cd8a	1	272	8.40	0.004
defbl2	1	273	9.82	0.002
lyz	1	274	16.23	< 0.0005
il1r-like	1	274	10.37	0.001
tbk1	1	273	6.39	0.012
gpx4a	1	274	13.56	< 0.0005

[†]Models were of the form:

Gene expression variable$_i$ = β_0 + $\boldsymbol{\beta_1}$ **habitat** + β_2 time (month) + β_3 sex + β_4 length + $\beta_5$condition + β_6 *Schistocephalus* (presence/absence) + Ɛ

A term for mean temperature in the 14 days before sampling was also included for genes (*defbl2* and *tbk1*) where temperature manipulation amongst mesocosms indicated that there was a significant effect of temperature (see main text Material and methods).

Table S1b. Significant results for the effect of habitat × time (month) on the expression of immune-associated genes in LMs†; considering only cases with a significant main effect of habitat in a main effects-only model.

**Gene**	**df1**	**df2**	***F***	***P***
igmh	10	260	2.82	0.002
cd8a	10	258	1.87	0.05
defbl2	10	259	4.34	< 0.0005
lyz	10	260	2.63	0.005
il1r-like	10	260	2.86	0.002

†Models were of the form:

Gene expression variable = β_0 + β_1 habitat + β_2 time (month) + β_3 sex + β_4 length + $\beta_5$condition + β_6 *Schistocephalus* (presence/absence) + β7 **habitat × time (month)** + Ɛ

For *defbl2* a term for mean temperature in the 14 days before sampling was also included, as temperature manipulation amongst mesocosms indicated that there was a significant effect of temperature (see main text Material and methods).
