# Supplementary material for: Changing expression of vertebrate immunity genes in an anthropogenic environment: a controlled experiment
Source: BMC Evol Biol. 2016 Sep 1;16(1):175. doi: 10.1186/s12862-016-0751-8 (PMC5009682; doi:10.1186/s12862-016-0751-8)
Supplement: Additional file 3: Table S2. — Differing gene expression variance in wild and mesocosm fishes. (PDF 136 kb) [file 12862_2016_751_MOESM3_ESM.pdf]

Table S2. Differing gene expression variance in wild and mesocosm fishes (for individual genes): results of significant Levene's tests

Gene	*P* (all mesocosms)	*P* (unheated mesocosms)
defbl2	<0.0005	0.023
foxp3b	0.006	0.052
gpx4a	<0.0005	< 0.0005
igzh	0.023	0.008
lyz	<0.0005	< 0.0005
il1r-like	0.008	0.015
